# Ocean Net Heat Flux Influences Seasonal to Interannual Patterns of Plankton Abundance

**DOI:** 10.1371/journal.pone.0098709

**Published:** 2014-06-11

**Authors:** Tim J. Smyth, Icarus Allen, Angus Atkinson, John T. Bruun, Rachel A. Harmer, Robin D. Pingree, Claire E. Widdicombe, Paul J. Somerfield

**Affiliations:** Plymouth Marine Laboratory, Plymouth, Devon, United Kingdom; University of Connecticut, United States of America

## Abstract

Changes in the net heat flux (NHF) into the ocean have profound impacts on global climate. We analyse a long-term plankton time-series and show that the NHF is a critical indicator of ecosystem dynamics. We show that phytoplankton abundance and diversity patterns are tightly bounded by the switches between negative and positive NHF over an annual cycle. Zooplankton increase before the transition to positive NHF in the spring but are constrained by the negative NHF switch in autumn. By contrast bacterial diversity is decoupled from either NHF switch, but is inversely correlated (r = −0.920) with the magnitude of the NHF. We show that the NHF is a robust mechanistic tool for predicting climate change indicators such as spring phytoplankton bloom timing and length of the growing season.

## Introduction

The air-sea exchange of heat, critical for regulating the Earth's climate [1], is also related to the turbulence structure of the upper ocean [2] which in turn controls plankton dynamics [3]. As phytoplankton form the base of the marine food web, an understanding of their population dynamics, including species succession [4], is important for determining whole ecosystem trajectories. The spring bloom is manifested as a dramatic increase in the phytoplankton standing stock over a relatively short period of time [5]. This phenomenon is caused by a combination of increasing solar irradiance, abundant surface layer nutrients and a stabilizing water column [6]–[8] occurring in concert.

The idea of a critical depth above which the mixed layer shoals during the spring, marking the start of the bloom, has become synonymous with Sverdrup [9]. Recently, however, several authors have shown that the start of the spring bloom can precede the critical depth being reached by several weeks [3], [10] leading to alternative theories being posited. These include: (i) the dilution-recoupling hypothesis [7] where the mixed layer depth deepening during winter causes a dilution between phytoplankton and their predators allowing an initial increase in phytoplankton biomass, before recoupling them as the mixed layer depth shoals; (ii) the critical turbulence hypothesis [3], [6] where the spring bloom is triggered by a reduction in air-sea heat fluxes leading to a weakening of the turbulence in the mixed layer allowing phytoplankton sufficient residence time in a lit layer replete with nutrients to bloom and; (iii) the eddy driven hypothesis [11] where deep oceanic eddies cause initial stratification, rather than a springtime warming of the sea surface, favourable for the growth and multiplication of phytoplankton.

For an appraisal of the start of the spring bloom it is important to realise the conceptual difference between the mixed layer depth (MLD) and the depth of the layer which supports initial phytoplankton growth, which may be much shallower than this. The MLD requires a definition: this is often arbitrarily set to the depth at which there is a pre-determined (e.g. −0.2°C) change in temperature [12].

Due to winter mixing, the MLD in the Bay of Biscay can be >500 m in March [13]. However, the spring bloom is initiated within a surface euphotic layer that may only be 10 m deep with temperature differences as little as 0.1°C. This is unrelated to, and certainly not discriminated by, a MLD definition of −0.2°C, which may still be >200 m at the height of the bloom [14].

Similarly, this observation is repeated on the NW European continental shelf [15], where the spring bloom is initiated in a mixed (to 0.01°C) water column, but with a stabilised surface layer of 0.1°C. From the two examples given above, using a MLD appraisal of conditions will lead to the conclusion that significant production takes place in the winter in temperate seas before any surface stabilisation.

To overcome the need for arbitrary MLD definitions, flux methods have previously been employed to determine the start of the productive season [16] with a balance (R) of potential energy (PE) and turbulent kinetic energy (TKE) production controlling near surface stabilisation:
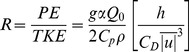
(1)where g is the acceleration due to gravity, α the volume coefficient of expansion, C_p_ the specific heat at constant pressure, Q_0_ the surface heat flux, ρ the density of sea water, C_D_ the drag coefficient, h the water depth and |u| the magnitude of the instantaneous tidal stream velocity, u, with the overbar denoting the average value over one cycle. In this paper we use a limit of this approach, namely that the net heat flux (NHF) at the surface (Q_0_) needs only to be a positive quantity for stabilisation.

The focus of this study on plankton bloom dynamics is centred on a tidally influenced, shelf seas time-series station (L4: 50° 15′N, 4° 13′W; depth  = 54 m) situated in the western English Channel (Figure S1). We use long time-series, weekly resolved phytoplankton and zooplankton data to study relationships between plankton population dynamics, including biodiversity, and modelled NHF [17].

## Materials and Methods

### Ethics statement

No specific permissions were required for the plankton sampling at station L4 (50° 15′N, 4° 13′ W), and the field studies did not involve endangered or protected species.

### Net Heat Flux

The air-sea flux of heat is governed by four processes: shortwave radiation from the sun (Q_SW_), outgoing longwave radiation from the sea surface (Q_LW_), sensible heat transfer resulting from air-sea temperature differences (Q_SH_), and the latent heat transfer carried by evaporation of sea surface water (Q_LH_). Direct observations (e.g. using ships and buoys) of the air-sea heat flux are rare because of the difficulty in taking such measurements. However, it is possible to determine the four components of the air-sea heat flux using bulk parameterizations which are a function of surface meteorological and oceanographic variables. We used the Woods Hole Oceanographic Institution air-sea exchange Matlab tools [18] to determine Q_SW_, Q_LW_, Q_SH_ and Q_LH_ [17], all in units of Wm^−2^. The meteorological parameters used to run the heat flux model (HFM) were obtained from the European Centre for Medium Range Weather Forecasting (ECMWF) ERA-40 and Operational analyses and extracted for a gridpoint centred on 50° N, 4° W. These parameters were: air temperature (T_a_ in °C), dew point (T_d_ in °C), wind-speed at 10 m (U_10_ in m s^−1^), cloud fraction (CF where 0: clear; 1: overcast) and atmospheric pressure (P in mb). Sea surface temperatures (T_s_ in °C) measured at stations L4 and E1 (50° 02′N, 4° 22′W) were used to run the HFM over different periods overlapping with the ECMWF data availability (1958–2011). Station L4 has available T_s_ between 1988 and 2011, whereas station E1 has records of T_s_ between 1903 and 2011. Q_SW_ was calculated as a function of date and position with correction for CF [19]; Q_LW_ as a function of T_a_, T_s_, T_d_, CF using the Berliand bulk formula [20]. Both Q_SH_ and Q_LH_ were calculated as a function of T_a_, T_s_, T_d_, CF, P and U_10_. The sum of all four components results in the net heat flux (NHF) with the sign convention of positive NHF being into the water column used throughout.

The HFM was run between 1988 and 2010 for L4 and from 1958–2011 for E1, after the input T_s_ had been interpolated from the weekly sampling interval onto a daily time resolution grid. This was to enable the NHF to be calculated on the same time resolution as the available (daily) meteorological values. The day of each individual year where the NHF became positive, and remained positive for more than seven days, was determined. A period of seven days was chosen for two reasons. Firstly, studies of phytoplankton succession rate show that it requires between two and four generations of the ascendant species to alter the community structure. This equates to between 5 and 15 days [21], given the rate at which phytoplankton can double their numbers, which is likely to be temperature-dependent. Secondly, routine L4 sampling takes place on a weekly basis, so the granularity of the time-series is of order seven days before interpolation. The choice of a seven day period had the added benefit of ensuring that the sign of the NHF is properly established rather than being fleeting or ephemeral. On calm days, even in winter, if there are clear skies, solar insolation can be sufficient to ensure the NHF is positive. The day of NHF switching back to negative towards the end of the year was also determined and the number of days between the two NHF switching events (negative to positive and positive to negative) was calculated for each year.

### Biological sampling

Key ecosystem parameters have been sampled in the western English Channel for over a century [22], [23]. The weekly phytoplankton samples (since 1992: see Table S1 for species list) were taken at a depth of 10 m using a 10 L Niskin bottle at station L4. A 200 mL subsample was then removed from the bottle and immediately fixed with 2% (final concentration) Lugol's iodine solution [24]. A second 200 mL sub-sample was also taken and preserved with neutral formaldehyde for the enumeration of coccolithophores. The samples were stored in cool, dark conditions until taxonomic analysis at the Plymouth Marine Laboratory (PML) using light microscopy [25]. Samples for zooplankton have been collected on a weekly basis since 1988 (see Table S2 for species list), using a vertical net haul from 50 m depth to the surface using a WP2 net with a mesh-size of 200 µm and mouth area of 0.25 m^2^ [26]. Two hauls are taken and the samples preserved and stored in 5% formalin. These are then sub-sampled, counted and identified using light microscopy at PML [27]. Bacterial abundance data (2003–2008) were generated by extracting nucleic acids from 5 L seawater samples collected from the surface and filtered immediately through a 0.22 µm Sterivex cartridge (Millipore), which was stored at −80°C. DNA was isolated from each sample and then stored at −20°C prior to 454-pyrosequencing [28].

### Statistical analyses

The biological datasets were linearly interpolated onto a daily grid from the original weekly sampling intervals for the entire length of the time-series. This daily grid was then shifted in time with t = 0 representing the serial day number of the NHF becoming positive for each individual year (Figure S2). This puts the biological signal for each individual year into a common frame of reference. Once each year had been time-shifted to day zero, the biological signal for each day for all years was smoothed, using a median filter, to remove isolated high or low values. This reduces the multiple annual time-series to a single daily value (for each individual biological time-series) allowing the numerical analyses described below to be performed.

The bloom onset date was defined as the first day of rapid increase in total phytoplankton abundance which is then followed for more than seven days by values greater than the winter baseline i.e. a near step-wise change with a definite edge. This was numerically determined from the time-series data using an Auto-Regressive Integrated Moving Average (ARIMA) transfer function model [29]. The onset edge was estimated through use of a ‘ragged-step’ intervention function and uses the Akaike Information Criterion (AIC) to identify the relevant order of the ARIMA model to use in this setting. That specific model for the onset day is then estimated using maximum likelihood optimisation. This algorithm was coded in the R-statistical language.

The number of taxa (S) represented in the various samples was extracted from the phytoplankton, holozooplankton, merozooplankton and bacterial datasets. This was to give a measure of the biodiversity for the various planktonic functional groups and to examine plankton dynamics, as a function of heat flux, over a range of trophic levels.

Using the 54 complete year time-series of heat flux, positive NHF and negative NHF onset dates at station E1 (1958–2011) were determined. A spectral analysis approach was used to identify the regular repeating harmonic terms in the time-series signal [29]. The contribution of the harmonic and trend components to the signal were then estimated through the Box-Jenkins time-series model [30] approach.

## Results

**Figure 1:**
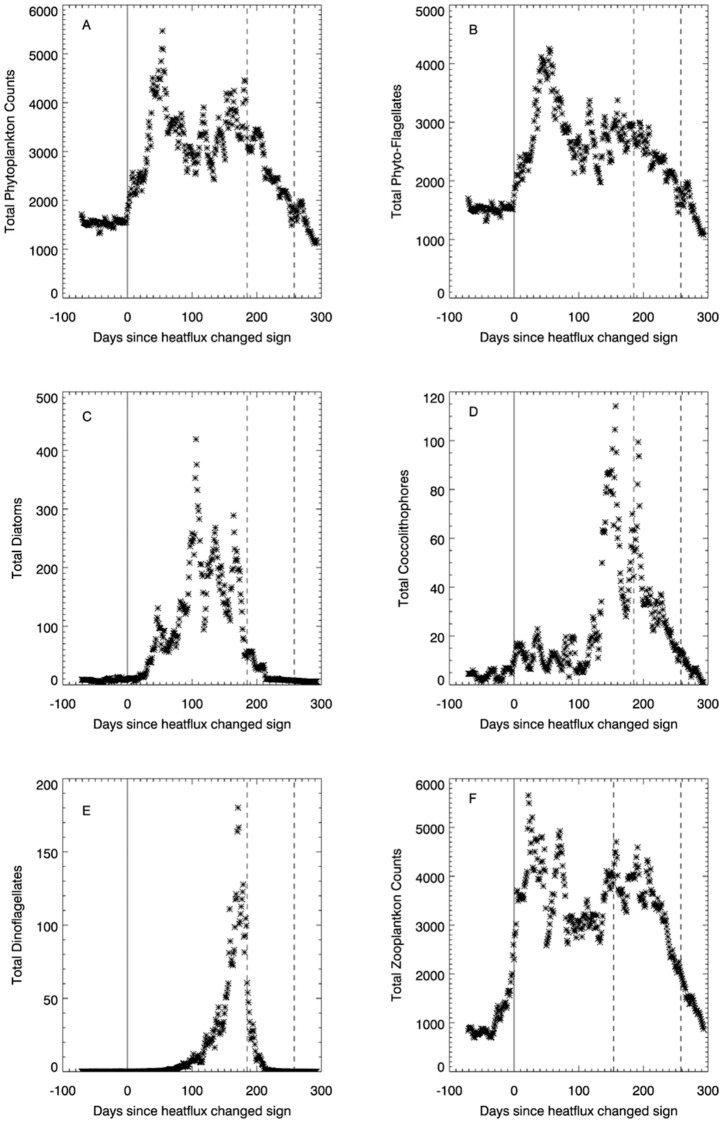
Entire time-normalized series at station L4 with a median filter applied to the phytoplankton (1992–2010) and zooplankton (1988–2010) abundance data (units: cells mL^−1^, individuals m^−3^, respectively). Phytoplankton species are aggregated as (A) total; (B) phytoflagellates; (C) diatoms; (D) coccolithophores and (E) dinoflagellates. Zooplankton are aggregated as (F) total zooplankton. The solid line indicates day zero, where the NHF switches from negative to positive and remains positive for more than 7 days. The two dashed vertical lines shown in all the panels indicate the minimum and maximum number of days after the positive NHF switch that the NHF returns to being negative (for more than 7 days).

Figure 1 shows that when the NHF switches to positive, the total phytoplankton abundance increases rapidly, reaching peak abundance around 40 days later. The median NHF positive switch is serial day 71 (11 March, range: 19 February – 21 April) for L4. Using objective time-series statistical techniques [29], the first increase in the abundance of total phytoplankton was calculated to be day 70. The commencement of the rapid increase in phytoplankton abundance (Figure 1A.), precedes the maximum abundance by between 40 and 50 days (serial day 111–121) which is consistent with previously observed peak bloom timings in the western English Channel [31]. Total phytoplankton abundance (Figure 1A.) is initially dominated by species of phytoflagellates (Figure 1B. – e.g. *Phaeocystis*). This is followed by multiple peaks of diatoms (Figure 1C.) starting around 30 days after the NHF switch. Coccolithophores (Figure 1D.) and dinoflagellates (Figure 1E.) dominate later in the season. Zooplankton abundance (Figure 1F.) starts to increase (day 41) before either the NHF positive switch or the increase in total phytoplankton.

From Figure 1 it appears that the length of the growing season is constrained by the switch of sign from negative to positive NHF in the spring and the reverse in the autumn (dashed lines). The latter occurs between 185 (1999) and 258 (1997) days after the NHF positive switch at L4, for the period studied here (1988–2010). We therefore use the period between these switching events as a proxy for the length of the growing season.

**Figure 2:**
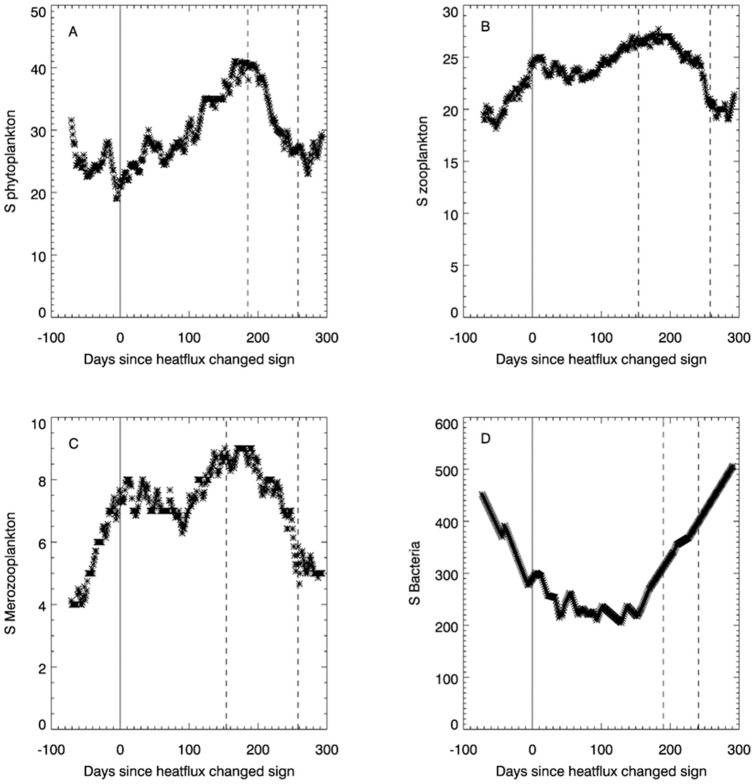
Median filtered daily interpolated biodiversity (species richness –S) data for (A) phytoplankton; (B) zooplankton; (C) merozooplankton and (D) bacteria relative to the change in sign for the NHF. The switch to negative heat flux (relative to the switch to positive and shown as the minimum and maximum range) is shown later in the season around 185–260 days afterwards, represented by the pair of dashed lines.

Figure 2 shows the temporal evolution of plankton species richness (phytoplankton, holozooplankton, meroplankton and bacteria). The number of species (S) of phytoplankton (Figure 2A.) reaches a minimum of approximately 20 at the onset of the spring bloom, peaking at around 40 just before the NHF negative switch. Following this the number of species drops to around 25. The variability of phytoplankton species biodiversity with respect to the NHF differs markedly in comparison to other plankton types. For phytoplankton (Figure 2A.) the spring bloom is characterized by a smaller number of dominant species that are ideally suited to exploit the particular conditions of temperature, light and nutrients. Both holozooplankton (Figure 2B.) and merozooplankton (Figure 2C.) show an early season peak coincident with the NHF positive switch, gradually increasing later in the season before reducing markedly when the NHF switches to negative.

**Figure 3:**
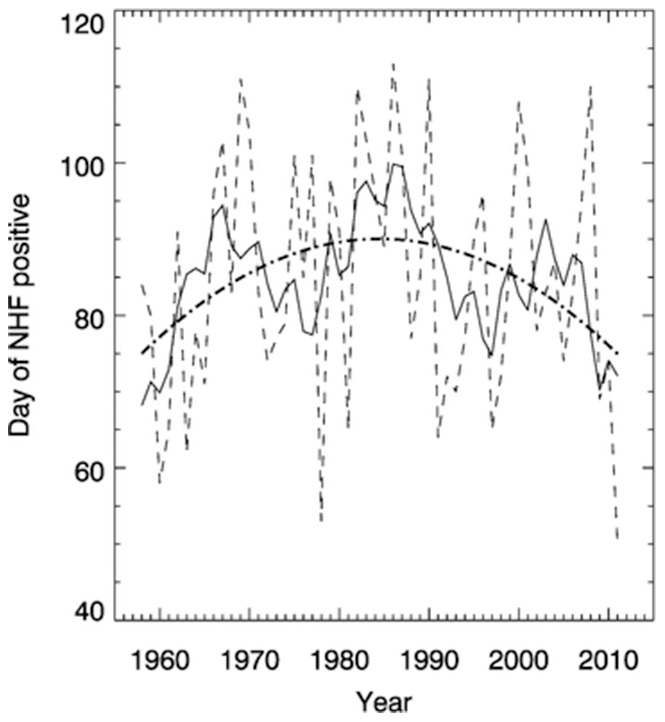
Onset of spring bloom day, determined using the NHF calculation (dashed line), between 1958 and 2011 at station E1. The solid line represents a spectral analysis of the NHF calculated, day of bloom onset, time-series using a Box-Jenkins [30] approach. The dash-dot line is a statistically significant (p = 0.004; r = 0.294) long-term cycle.

Station E1 provides a longer time-series of sea temperature to use in the calculations of NHF (Figure 3). This shows significant (*p* = 0.002) evidence of a 19 year repeating cycle in the timing of the change in sign of the NHF, with an envelope around this smooth oscillation of ±10 days. Based on this 19 year harmonic of the time-series, the spring bloom timing changes on average by two days per year. This shift represents smooth background climatic variability; interannual variability is captured by the dashed line in Figure 3 (range  = 63 days). There is also evidence of a longer term cycle (p = 0.004), represented by the dash-dot quadratic curve in Figure 3, which on average contributes to a changing start of spring bloom date by 0.4 days per year.

## Discussion

Figure 1 clearly shows a strong link between the change to positive NHF and the start of the spring phytoplankton bloom indicating that stabilization of the water column gives phytoplankton [32] a window of opportunity to proliferate. The advantage of using the NHF is that it can be calculated using surface-only abiotic data: these are readily available from a combination of in situ observations, meteorological model analyses and satellite platforms. The NHF predictor can therefore be applied to real-time global ocean observations as well as to seasonal-decadal scale forecasts based on climate change projections.

Figure 1F, perhaps surprisingly, suggests that zooplankton have life strategies which allow them to pre-empt the spring bloom giving them a competitive edge at the start of the season. However, there are likely to be other mechanisms at work. The early spring increase in zooplankton is caused partly by merozooplankton such as barnacle larvae, whose spawning cues are likely different. The background levels of chlorophyll-a concentration in the western English Channel during the winter are around 0.5 mg m^−3^ [23] and support zooplankton reproduction at L4 year-round. During the narrow time (30 d) window between the increase in zooplankton abundance and NHF positive, grazing control by micro and mesozooplankton may be in operation. The reduction in turbulence, indicated by NHF becoming positive, will allow phytoplankton such as *Phaeocystis* (colonies) and diatoms (chains) to break through the grazing control “loophole” [33], due to increased residence time in the well-lit layer of the water column.

Towards the autumn the switch to negative NHF appears to be an important cut-off for phytoplankton species that require thermal stratification to thrive, such as coccolithophores [34] and dinoflagellates such as *Karenia mikimotoi* [25]. Dinoflagellates are likely to be favoured later in the season because, as the NHF becomes less positive, the exchange of nutrients across a weakening thermocline increases [35]. It is important to note that the mechanisms being invoked here are purely abiotic: it is likely other, as yet unquantified, loss mechanisms such as viral infection and parasitism, sinking and grazing play a part in the seasonal succession.

The NHF seems to be less of a factor for zooplankton (Figure 2B.), as they are more strongly governed by temperature and are able to easily migrate through the water column [36]. How prolific they are will depend on the amount and quality of available food.

**Figure 4:**
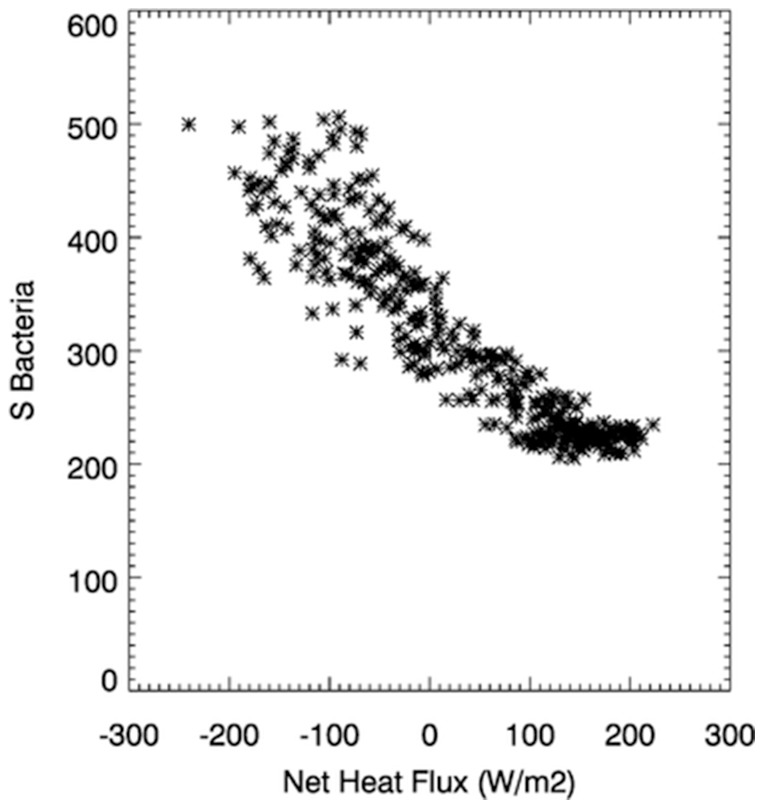
Bacterial diversity versus the magnitude of NHF for station L4 (r = −0.920). Each individual data point represents a daily average for the period 2003–2008.

Bacterial diversity (Figure 2D.) is disconnected completely from the switch from negative to positive NHF. Previous studies at L4 [28] concluded that bacterial diversity (Figure 2D.) is related to daylength (minimum/maximum at summer/winter solstices). Figure 4 shows that bacterial diversity is strongly negatively correlated with the magnitude of the NHF (r = −0.920). This implies that bacterial diversity is favoured in highly turbulent conditions, providing a more plausible mechanistic explanation than a simple relationship with daylength. To test the wider applicability of this finding other high temporal (weekly) resolution bacterial time-series data would be required, from stations where the turbulent structure of the upper mixed layer is not necessarily in phase with the annual solar cycle. A weekly sampling routine should be adequate to describe seasonal variability in the bacterial diversity.

Figure S3 implies that the change from negative to positive (spring) and positive to negative (autumn) NHF are decoupled in regions of seasonal stratification i.e. an early onset of the spring bloom does not imply a late switch of NHF from positive to negative later in the year. Indeed there is no correlation (r = −0.01) between NHF positive and NHF negative date. Rather it seems that the length of the growing season is constrained by how early in the year positive NHF is established, i.e. there is more variability in spring bloom onset date (figure 3, dashed line) than the breakdown in stratification date in autumn (figure S4, dashed line). Mean NHF positive date (1958–2011) is 84.8±16.0 whereas mean NHF negative date for this period is 287.3±15.3. This is because the upper mixed layer is both wind mixed and convectively mixed as a result of surface heat flux, particularly at night [37]. The invariant factor in both onset and breakdown is daylight hours: these rapidly increase, and therefore promote positive NHF, in the spring and; rapidly decrease, and therefore promote negative NHF, in the autumn. Positive NHF is more difficult to establish in the spring than negative NHF in the autumn because of seasonal stratification at stations L4 and E1. In the spring, the water column is well mixed, so to promote NHF all the positive heat flux mechanisms (i.e. warmer atmosphere than sea-surface, reduction in wind, increasing levels of solar insolation) need to be acting in the same direction. A small perturbation to this, such as strong winds or a prolonged cold, cloudy period, will delay a switch to positive NHF and, as a consequence, thermal stratification. At the beginning of autumn, the top 10–15 m of the water column are thermally stratified, and because of the larger thermal heat capacity of water compared with air, as the nights get longer the tendency will be for all negative heat flux mechanisms to act in the same direction. This gives a more abrupt change in sign to negative NHF and hence a more distinct and invariant end point to the growing season (Figure S3).

One of the limitations of using a Eulerian time-series station such as L4, is that it does not allow the significant time-series noise to be resolved into local process and spatial patchiness components [38]. For such a partitioning to be made, fine scale spatial surveying would be required to smooth out the time-series. From the evidence of local, short duration Lagrangian studies, the effect of tides at station E1 tend to advect the water masses around in an ellipse, with the net result of little overall translational movement. At L4, there is a tendency for water masses to move in an east-west along-shore direction, with the added complication of episodic inputs from the River Tamar in terms of buoyancy, nutrient concentrations and material loading. However, the tides and the shallowness of L4 tend to damp variability [38].

The nineteen year repeating cycle in the timing of the change in sign of the NHF corresponds very closely to the 18.6 year tidal modulation period [39]. This shows the importance of the tides, probably in breaking down marginal stratification, for explaining seasonal changes in the abundance of phytoplankton [15].

The long-term quadratic temporal component could also be related to the Atlantic Multidecadal Oscillation (AMO), which has a periodicity of 65–70 years [40], [41]; the AMO has a direct influence upon the North Atlantic Oscillation, which has previously been postulated to control timing of the spring bloom [42]. It is important to take account of both the tidal and large scale climatic oscillation harmonics when looking for evidence of background climate change. Indeed when both the tidal and long-term quadratic components are acting in phase, this can result in the date of the NHF advancing (or retreating) by 2.4 days per year. The end of the growing period (Figure S4) does not exhibit a long-term trend, but has an observable dual-frequency 20 year repeating pattern. With this repeating pattern, we note that over this 54 year period there are three events, of 5 years duration, which may indicate the emergence of a long-term increasing variability in the end day of the growing period. These statistical techniques therefore allow us to disentangle patterns of natural variability from any background climate change signal in the bloom onset and length of growing season.

## Conclusions

We have demonstrated that the NHF provides a robust and powerful tool to determine essential climate change indicators such as the onset of the spring bloom and length of the growing season. The onset of the spring phytoplankton bloom has been shown to coincide with a switch from negative to positive NHF. At the end of the growing season, the switch back to negative NHF has been shown to control the succession of later blooming species of phytoplankton, effectively providing a cut-off to their proliferation. We have shown that zooplankton abundance pre-empts the NHF switch in spring, and that bacterial diversity patterns are structurally different to both phytoplankton and zooplankton. The latter suggests a tighter control of turbulence upon the diversity of the smallest organisms rather than a NHF switching mechanism which impacts upon phytoplankton diversity.

Using statistical tools on long-term ecological time-series data, we have been able to show the impact of a variety of physical and large scale climate forcing mechanisms, such as the 18.6 year tidal cycle and possibly the 65–70 year AMO, upon the length of the growing season.

As NHF can be calculated using surface only abiotic data, its predictive power can be applied to the global ocean using a combination of in situ observations, meteorological model analyses and satellite platforms. The NHF predictor can therefore be applied to real-time global ocean observations as well as to seasonal to decadal forecasts based on climate change projections.

## Supporting Information

Figure S1 **Map showing the locations of stations L4 (50° 15′N, 4° 13′W) and E1 (50° 02′N, 4° 22′W).**
(TIFF)

Figure S2 **Daily interpolated phytoplankton abundance data for individual years 1992–2010 relative to the change in sign for the NHF.** The figure shows the inherent difficulty in working with quantitative biological datasets as there is a large degree of inter- and intra-annual variability. However a general pattern of increasing phytoplankton abundance around the time of the NHF switch emerges. It could be argued that biomass should be used rather than numerical counts, as abundances of smaller phytoplankton (e.g. phytoflagellates), which contain less carbon than larger phytoplankton (e.g. diatoms), may be present in disproportionately large numbers. When the identical statistical time-series analysis (see text and Figure 1) was repeated for total phytoplankton biomass, the first increase was calculated to be on day 69.
(TIFF)

Figure S3 **Length of growing season, calculated using the period of time between NHF positive and switching back to negative, as a function of NHF positive day.** Calculations are for the period 1958–2011 at station E1. Fitted line has r = −0.73.
(TIFF)

Figure S4 **Day of NHF negative (dashed line), between 1958 and 2011 at station E1, indicative of the end of the growing season.** The solid line represents a spectral analysis of the calculated NHF time-series using a Box-Jenkins [30] approach.
(TIFF)

Table S1 **Station L4 Phytoplankton and Microzooplankton Taxa List.**
(DOCX)

Table S2 **Station L4 Zooplankton Taxa List.**
(DOCX)
