# Supplementary figures and images for: Ocean Net Heat Flux Influences Seasonal to Interannual Patterns of Plankton Abundance
Source: PLoS One. 2014 Jun 11;9(6):e98709. doi: 10.1371/journal.pone.0098709 (PMC4053316; doi:10.1371/journal.pone.0098709)

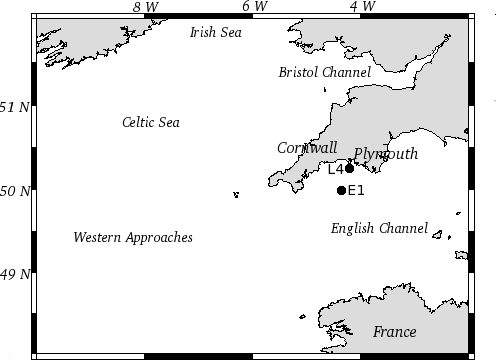

Supplement: Figure S1 — Map showing the locations of stations L4 (50° 15′N, 4° 13′W) and E1 (50° 02′N, 4° 22′W). (TIFF) [file pone.0098709.s001.tiff]

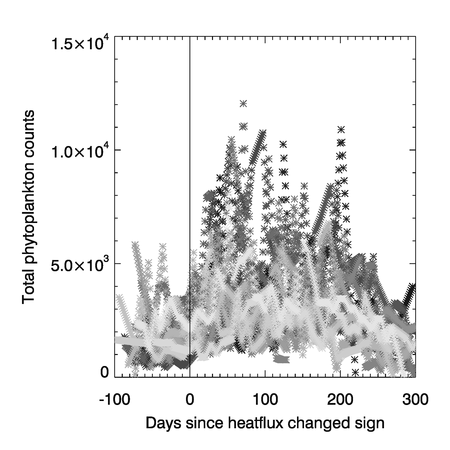

Supplement: Figure S2 — Daily interpolated phytoplankton abundance data for individual years 1992–2010 relative to the change in sign for the NHF. The figure shows the inherent difficulty in working with quantitative biological datasets as there is a large degree of inter- and intra-annual variability. However a general pattern of increasing phytoplankton abundance around the time of the NHF switch emerges. It could be argued that biomass should be used rather than numerical counts, as abundances of smaller phytoplankton (e.g. phytoflagellates), which contain less carbon than larger phytoplankton (e.g. diatoms), may be present in disproportionately large numbers. When the identical statistical time-series analysis (see text and Figure 1) was repeated for total phytoplankton biomass, the first increase was calculated to be on day 69. (TIFF) [file pone.0098709.s002.tiff]

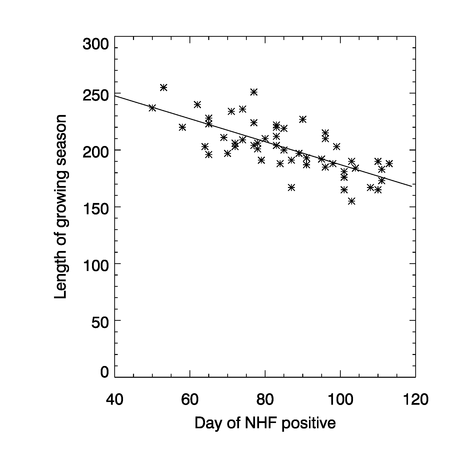

Supplement: Figure S3 — Length of growing season, calculated using the period of time between NHF positive and switching back to negative, as a function of NHF positive day. Calculations are for the period 1958–2011 at station E1. Fitted line has r = −0.73. (TIFF) [file pone.0098709.s003.tiff]

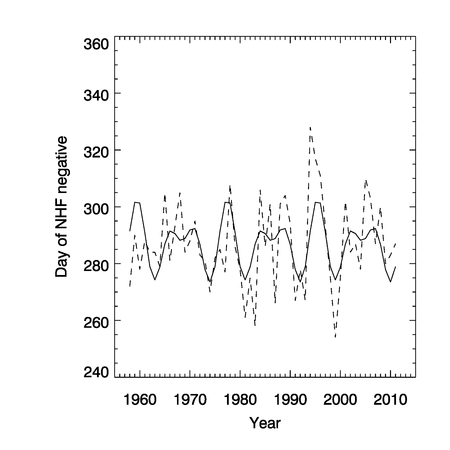

Supplement: Figure S4 — Day of NHF negative (dashed line), between 1958 and 2011 at station E1, indicative of the end of the growing season. The solid line represents a spectral analysis of the calculated NHF time-series using a Box-Jenkins [30] approach. (TIFF) [file pone.0098709.s004.tiff]
