# Supplementary material for: Ocean Net Heat Flux Influences Seasonal to Interannual Patterns of Plankton Abundance
Source: PLoS One. 2014 Jun 11;9(6):e98709. doi: 10.1371/journal.pone.0098709 (PMC4053316; doi:10.1371/journal.pone.0098709)
Supplement: Table S1 — Station L4 Phytoplankton and Microzooplankton Taxa List. (DOCX) [file pone.0098709.s005.docx]

## Table S1: Station L4 Phytoplankton and Microzooplankton Taxa List

| TAXA | WORMS ID | | Authority |
| --- | --- | --- | --- |
| TOTAL PHYTOPLANKTON | N/A | |  |
| TOTAL DIATOMS | N/A | |  |
| *Achnanthes longipes* | 156533 | | C.Agardh, 1824 |
| *Actinocyclus* | 148944 | | Ehrenberg, 1837 |
| *Actinoptychus senarius* | 148948 | | (Ehrenberg) Ehrenberg, 1843 |
| *Asterionellopsis glacialis* | 149139 | | (Castracane) Round, 1990 |
| *Bacillaria paxillifera* | 558243 | | (O.F.Müller) T.Marsson, 1901 |
| *Bacteriastrum furcatum* | 164110 | | Shadbolt, 1854 |
| *Trigonium alternans* | 699394 | | (J.W.Bailey) A.Mann, 1907 |
| *Brockmanniella brockmannii* | 149137 | | (Hustedt) Hasle, Stosch & Syvertsen, 1983 |
| *Odontella mobiliensis* | 164116 | | (J.W.Bailey) Grunow, 1884 |
| *Odontella sinensis* | 149095 | | (Greville) Grunow, 1884 |
| *Cerataulina pelagica* | 149619 | | (Cleve) Hendey, 1937 |
| *Chaetoceros* | 148985 | | Ehrenberg, 1844 |
| *Chaetoceros affinis* | 149241 | | Lauder, 1864 |
| *Chaetoceros anastomosans* | 149292 | | Grunow, 1882 |
| *Chaetoceros f. borealis* | 149124 | | J.W.Bailey, 1854 |
| *Chaetoceros brevis* | 149291 | | F.Schütt, 1895 |
| *Chaetoceros compressus* | 149129 | | Lauder, 1864 |
| *Chaetoceros costatus* | 149289 | | Pavillard, 1911 |
| *Chaetoceros curvisetus* | 149221 | | Cleve, 1889 |
| *Chaetoceros danicus* | 149120 | | Cleve, 1889 |
| *Chaetoceros debilis* | 149219 | | Cleve, 1894 |
| *Chaetoceros decipiens* | 149126 | | Cleve, 1873 |
| *Chaetoceros densus* | 149121 | | (Cleve) Cleve, 1899 |
| *Chaetoceros didymus* | 149122 | | Ehrenberg, 1845 |
| *Chaetoceros eibenii* | 160521 | | Grunow, 1882 |
| *Chaetoceros externus* | 160522 | | Gran, 1897 |
| *Chaetoceros filiformis* | 178194 | | Meunier, 1910 |
| *Chaetoceros fragilis* | 149173 | | Meunier, 1910 |
| *Chaetoceros laciniosus* | 149228 | | F.Schütt, 1895 |
| *Chaetoceros lauderi* | 160523 | | Ralfs, 1864 |
| *Chaetoceros peruvianus* | 178185 | | Brightwell, 1856 |
| *Chaetoceros protuberans* | 163055 | | H.S.Lauder, 1864 |
| *Chaetoceros radicans* | 163112 | | F.Schütt, 1895 |
| *Chaetoceros resting spores* | 148985 | |  |
| *Attheya septentrionalis* | 162823 | | (Østrup) R.M.Crawford, 1994 |
| *Chaetoceros similis* | 149127 | | Cleve, 1896 |
| *Chaetoceros simplex* | 149294 | | Ostenfeld, 1901 |
| *Chaetoceros socialis* | 149123 | | H.S.Lauder, 1864 |
| *Chaetoceros teres* | 149125 | | Cleve, 1896 |
| *Chaetoceros tortissimus* | 163161 | | Gran, 1900 |
| *Chaetoceros wighamii* | 160524 | | Brightwell, 1856 |
| *Chaetoceros willei* | 156625 | | Grunow, 1897 |
| *Corethron pennatum* | 341496 | | (Grunow) Ostenfeld, 1909 |
| *Coscinodiscus asteromphalus* | 149274 | | Ehrenberg, 1844 |
| *Coscinodiscus centralis* | 149159 | | Ehrenberg, 1844 |
| *Coscinodiscus concinnus* | 148992 | | W.Smith, 1856 |
| *Coscinodiscus granii* | 149271 | | Gough, 1905 |
| *Coscinodiscus radiatus* | 149158 | | Ehrenberg, 1840 |
| *Coscinodiscus wailesii* | 156632 | | Gran & Angst, 1931 |
| *Dactyliosolen blavyanus* | 179786 | | (H.Peragallo) Hasle, 1975 |
| *Dactyliosolen fragilissimus* | 149310 | | (Bergon) Hasle, 1996 |
| *Delphineis* | 149179 | | G.W.Andrews, 1977 |
| *Detonula pumila* | 149647 | | (Castracane) Gran, 1900 |
| *Diploneis crabro* | 149396 | | (Ehrenberg) Ehrenberg, 1854 |
| *Ditylum brightwellii* | 149023 | | (T.West) Grunow, 1885 |
| *Ephemera planamembranacea* | 341542 | | (Hendey) Paddock, 1988 |
| *Eucampia zodiacus* | 149131 | | Ehrenberg, 1839 |
| *Fragilaria* | 149028 | | Lyngbye, 1819 |
| *Fragilariopsis* | 149313 | | Hustedt, 1913 |
| *Grammatophora* | 149335 | | Ehrenberg, 1840 |
| *Guinardia delicatula* | 149112 | | (Cleve) Hasle, 1997 |
| *Guinardia flaccida* | 149132 | | (Castracane) H.Peragallo, 1892 |
| *Haslea wawrikae* | 248063 | | (Husedt) Simonsen, 1974 |
| *Lauderia annulata* | 149135 | | Cleve, 1873 |
| *Leptocylindrus danicus* | 149106 | | Cleve, 1889 |
| *Leptocylindrus mediterraneus* | 149230 | | (H.Peragallo) Hasle, 1975 |
| *Leptocylindrus minimus* | 149039 | | Gran, 1915 |
| *Licmophora* | 149342 | | C.Agardh, 1827 |
| *Lioloma delicatulum* | 292728 | | (Cupp) Hasle, 1996 |
| *Lithodesmium undulatum* | 149322 | | Ehrenberg, 1839 |
| *Melosira* | 149042 | | C.Agardh, 1824 |
| *Navicula* | 149142 | | Bory de Saint-Vincent, 1822 |
| *Navicula distans* | 149143 | | (W.Smith) Ralfs, 1861 |
| Small Pennate | 149001 | |  |
| Pennate 30µm | 149001 | |  |
| Pennate 50µm | 149001 | |  |
| V. small Pennate | 149001 | |  |
| *Nitzschia sigmoidea* | 149604 | | (Nitzsch) W.Smith, 1853 |
| *Cylindrotheca closterium* | 149004 | | (Ehrenberg) Reimann & J.C.Lewin, 1964 |
| *Psammodictyon panduriforme* | 149217 | | (W.Gregory) D.G.Mann, 1990 |
| *Pseudo-nitzschia "delicatissima"* | 149153 | | (Cleve) Heiden, 1928 |
| *Pseudo-nitzschia "pungens"* | 160528 | | (Grunow ex Cleve) G.R.Hasle, 1993 |
| *Pseudo-nitzschia "seriata"* | 149152 | | (Cleve) H.Peragallo, 1899 |
| *Paralia sulcata* | 149055 | | (Ehrenberg) Cleve, 1873 |
| *Planktoniella sol* | 196815 | | (C.G.Wallich) Schütt, 1892 |
| *Pleurosigma* | 149181 | | W.Smith, 1852 |
| *Pleurosigma planctonicum* | 231883 | | Cleve-Euler, 1952 |
| *Podosira stelligera* | 149060 | | (J.W.Bailey) A.Mann, 1907 |
| *Proboscia alata* | 149168 | | (Brightwell) Sundström, 1986 |
| *Proboscia alata 5µm* | 149168 | | (Brightwell) Sundström, 1986 |
| *Proboscia truncata* | 248181 | | (G.Karsten) Nöthig & Ligowski, 1991 |
| *Rhizosolenia chunii* | 341502 | | Karsten, 1905 |
| *Rhizosolenia hebetata f. semispina* | 149071 | | (Hensen) Gran, 1905 |
| *Neocalyptrella robusta* | 345491 | | (G.Norman ex Ralfs) Hernández-Becerril & Meave del Castillo, 1997 |
| *Rhizosolenia setigera 5µm* | 149115 | | Brightwell, 1858 |
| *Rhizosolenia setigera 25µm* | 149115 | | Brightwell, 1858 |
| *Rhizosolenia imbricata 10µm* | 149116 | | Brightwell, 1858 |
| *Rhizosolenia imbricata 5µm* | 149116 | | Brightwell, 1858 |
| *Rhizosolenia imbricata 15µm* | 149116 | | Brightwell, 1858 |
| *Guinardia striata* | 149113 | | (Stolterfoth) Hasle, 1996 |
| *Guinardia striata* (large) | 149113 | | (Stolterfoth) Hasle, 1997 |
| *Rhizosolenia styliformis* | 149629 | | T.Brightwell, 1858 |
| *Proboscia alata (syn. f. gracillima)* | 149168 | | (Brightwell) Sundström, 1986 |
| *Roperia tesselata* | 149105 | | (Roper) Grunow ex Pelletan, 1889 |
| *Skeletonema costatum* | 149074 | | (Greville) Cleve, 1873 |
| *Meuniera membranacea* | 149145 | | (Cleve) P.C.Silva, 1996 |
| *Stephanopyxis palmeriana* | 231888 | | (Greville) Grunow, 1884 |
| *Helicotheca tamesis* | 157440 | | (Shrubsole) M.Ricard, 1987 |
| *Thalassionema nitzschioides* | 149093 | | (Grunow) Mereschkowsky, 1902 |
| *Thalassiosira punctigera* | 148936 | | (Castracane) Hasle, 1983 |
| *Thalassiosira cf angulata* | 148912 | | Cleve, 1873 |
| *Thalassiosira eccentrica* | 148922 | | (Ehrenberg) Cleve, 1903 |
| *Thalassiosira anguste-lineata* | 148914 | | (A.Schmidt) G.Fryxell & Hasle, 1977 |
| *Thalassiosira gravida* | 149102 | | Cleve, 1896 |
| *Thalassiosira gravida 15µm* | 149102 | | Cleve, 1896 |
| *Thalassiosira rotula* | 148942 | | Meunier, 1910 |
| *Thalassiosira subtilis* | 149101 | | (Ostenfeld) Gran, 1900 |
| *Thalassiosira 2µm* | 148912 | | Cleve, 1873 |
| *Thalassiosira 4µm* | 148912 | | Cleve, 1873 |
| *Thalassiosira 5µm* | 148912 | | Cleve, 1873 |
| *Thalassiosira 10µm* | 148912 | | Cleve, 1873 |
| *Thalassiosira 20µm* | 148912 | | Cleve, 1873 |
| *Thalassiosira 30µm* | 148912 | | Cleve, 1873 |
| *Thalassiosira 40µm* | 148912 | | Cleve, 1873 |
| *Thalassiosira 60µm* | 148912 | | Cleve, 1873 |
| *Thalassiothrix* | 157081 | | Cleve & Grunow, 1880 |
| *Tropidoneis* | 149518 | | Cleve, 1891 |
| *Nanoneis hasleae* | 248180 | | R.E.Norris, 1973 |
| Undet. Diatom | 148899 | |  |
| TOTAL DINOFLAGELLATES | 19542 | |  |
| *Amylax triacantha* | 110007 | | (Jörgensen) Sournia, 1984 |
| *Neoceratium furca* | 495659 | | (Ehrenberg) F.Gomez, D.Moreira & P.Lopez-Garcia, 2010 |
| *Neoceratium fusus* | 495660 | | (Ehrenberg) F.Gomez, D.Moreira & P.Lopez-Garcia, 2010 |
| *Neoceratium horridum* | 495666 | | (Gran) F.Gomez, D.Moreira & P.Lopez-Garcia, 2010 |
| *Neoceratium lineatum* | 495674 | | (Ehrenberg) F.Gomez, D.Moreira & P.Lopez-Garcia, 2010 |
| *Ceratium longipes* | 109964 | | (Bailey) Gran, 1902 |
| *Neoceratium macroceros* | 495678 | | (Ehrenberg) F.Gomez, D.Moreira & P.Lopez-Garcia, 2010 |
| *Neoceratium massiliense* | 495679 | | (Gourret) F.Gomez, D.Moreira & P.Lopez-Garcia, 2010 |
| *Neoceratium tripos* | 495702 | | (O.F.Müller) F.Gomez, D.Moreira & P.Lopez-Garcia, 2010 |
| *Dinophysis acuminata* | 109603 | | Claparède & Lachmann, 1859 |
| *Dinophysis acuta* | 109604 | | Ehrenberg, 1839 |
| *Dinophysis cf punctata* | 109462 | | Ehrenberg, 1839 |
| *Dinophysis sacculus* | 232261 | | Stein, 1883 |
| *Dinophysis tripos* | 109662 | | Gourret, 1883 |
| *Karenia mikimotoi* | 233024 | | (Miyake & Kominami ex Oda) G.Hansen & Ø.Moestrup, 2000 |
| *Gonyaulax* | 109519 | | Diesing, 1866 |
| *Gonyaulax digitale* | 110015 | | (Pouchet) Kofoid, 1911 |
| *Gonyaulax grindleyi* | 110023 | | Reinecke, 1967 |
| *Gonyaulax spinifera* | 110041 | | (Claparède & Lachmann) Diesing, 1866 |
| *Alexandrium tamarense* | 109714 | | (Lebour) Balech, 1995 |
| *Gonyaulax verior* | 110045 | | Sournia, 1973 |
| *Gymnodinium* | 109475 | | Stein, 1878 |
| *Gymnodinium pygmaeum* | 109825 | | Lebour, 1925 |
| *Heterocapsa* | 109540 | | Stein, 1883 |
| *Heterocapsa niei* | 233620 | | (Loeblich III) Morrill & Loeblich III, 1981 |
| *Heterocapsa triquetra* | 110153 | | (Ehrenberg) F.Stein, 1883 |
| *Mesoporos perforatus* | 232516 | | (Gran) Lillick, 1937 |
| *Micranthodinium* | 109511 | |  |
| *Prorocentrum balticum* | 110293 | | (Lohmann) Loeblich, 1970 |
| *Tryblionella compressa* | 447746 | | (J.W.Bailey) M.Poulin, 1990 |
| *Prorocentrum dentatum* | 110298 | | Stein, 1883 |
| *Prorocentrum micans* | 110303 | | Ehrenberg, 1834 |
| *Prorocentrum cordatum* | 232376 | | (Ostenfeld) Dodge, 1975 |
| *Prorcentrum triestinum* | 110316 | | J. Schiller, 1918 |
| *Scrippsiella trochoidea* | 110172 | | (Stein) Balech ex Loeblich III, 1965 |
| *Scrippsiella* (cyst) | 109545 | | Balech ex A.R.Loeblich III, 1965 |
| TOTAL COCCOLITHOPHORES | 115057 | |  |
| Unident. Coccolithophorid | 115057 | |  |
| Holococcolithophorid 14µm | 248178 | | L.Cros & J.R.Young, 2005 |
| Holococcolithophorid 8µm | 248178 | | L.Cros & J.R.Young, 2005 |
| *Acanthoica quattrospina* | 235802 | | Lohmann, 1903 |
| *Calciosolenia brasiliensis* | 555889 | | (Lohmann) J.R.Young, 2003 |
| *Braarudosphaera bigelowii* | 235922 | | (Gran & Braarud) Deflandre, 1947 |
| *Calyptrosphaera* | 235828 | | Lohmann, 1902 |
| *Coccolithus pelagicus* | 178600 | | (Wallich) J.Schiller, 1930 |
| *Coccolithus pelagicus f. hyalinus* | 555900 | | (K.R.Gaarder & J.Markali) A.Kleijne, 1991 |
| *Emiliania huxleyi* | 115104 | | (Lohmann) W.W.Hay & H.P.Mohler, 1967 |
| *Coronosphaera* | 235934 | | Gaarder, 1977 |
| *Syracosphaera molischii* | 236039 | | J.Schiller, 1925 |
| *Gephyrocapsa* | 235823 | | Kamptner, 1943 |
| *Rhabdolithes claviger* | 626382 | | (G.Murray & Blackman) Voeltzkow, 1902 |
| *Syracosphaera pulchra* | 235979 | | Lohmann, 1902 |
| *Umbellosphaera* | 235937 | | Paasche, 1955 |
| TOTAL PHYTO-FLAGELLATES | | N/A |  |
| Flagellate 2µm | 146220 | |  |
| Flagellates 5µm | 146221 | |  |
| Flagellates 15µm | 146221 | |  |
| *Corymbellus aureus* | 162519 | | J.C.Green, 1976 |
| *Cryptomonadaceae* | 17644 | | Ehrenberg, 1831 |
| *Dictyocha speculum* | 157260 | | Ehrenberg, 1839 |
| *Dictyocha fibula* | 157463 | | Ehrenberg, 1839 |
| *Dinobryon* | 157240 | | Ehrenberg, 1834 |
| *Eutreptiella* | 17657 | | A.da Cunha, 1914 |
| *Halosphaeria* | 100145 | | Linder, 1944 |
| *Meringosphaera* | 115075 | | Lohmann, 1932 |
| *Pterosperma* | 160595 | | Pochet, 1893 |
| *Pyramimonas* | 134529 | | Schmarda, 1849 |
| *Raphidophyceae* | 160581 | |  |
| *Phaeocystis* motile | 115088 | | Lagerheim, 1893 |
| *Phaeocystis pouchetii* | 115106 | | (Hariot) Lagerheim, 1896 |
| TOTAL MICROZOOPLANKTON |  | |  |
| TOTAL COLOURLESS DINOFLAGELLATES |  | |  |
| *Amphidinium crassum* | 109726 | | Lohmann, 1908 |
| *Amphidinium sphenoides* | 109754 | | WüIff, 1916 |
| *Amphidoma caudata* | 110005 | | Halldal, 1953 |
| *Cochlodinium* | 109474 | | Schütt, 1896 |
| *Dinophysis nasuta* | 232496 | | (Stein) Parke & Dixon, 1968 |
| *Diplopsalis* | 109515 | | Bergh, 1881 |
| *Colourless Gymnodinium* | 109475 | | Stein, 1878 |
| *Colourless Gymnodinium (small)* | 109475 | | Stein, 1878 |
| *Gyrodinium (small)* | 109476 | | Kofoid & Swezy, 1921 |
| *Gyrodinium(medium)* | 109476 | | Kofoid & Swezy, 1921 |
| *Gyrodinium (large)* | 109476 | | Kofoid & Swezy, 1921 |
| *Kofoidinium lebourae* | 233165 | | (Pavillard) F.J.R.Taylor, 1976 |
| *Gyrodinium spirale* | 109876 | | (Bergh) Kofoid & Swezy, 1921 |
| *Katodinium glaucum* | 109885 | | (Lebour) Loeblich III, 1965 |
| *Katodinium* | 109477 | | Fott, 1857 |
| *Nematodinium* | 604302 | |  |
| *Noctiluca scintillans* | 109921 | | (Macartney) Kofoid & Swezy, 1921 |
| *Oxytoxum* | 109528 | | Stein, 1883 |
| *Phalacroma rotundatum* | 156505 | | (Claparéde & Lachmann) Kofoid & Michener, 1911 |
| *Polykrikos schwarzii* | 109901 | | Bütschli, 1873 |
| *Pronoctiluca pelagica* | 109903 | | Fabre-Domergue, 1889 |
| *Protoperidinium* | 109553 | | Bergh, 1882 |
| *Protoperidinium bipes* | 110208 | | (Paulsen) Balech, 1974 |
| *Protoperidinium brevipes* | 110210 | | (Paulsen) Balech, 1974 |
| *Protoperidinium curtipes* | 110215 | | (Jörgensen) Balech, 1974 |
| *Protoperidinium depressum* | 110217 | | (Bailey) Balech, 1974 |
| *Protoperidinium divergens* | 110219 | | (Ehrenberg) Balech, 1974 |
| *Protoperidinium oceanicum* | 110240 | | (VanHöffen) Balech, 1974 |
| *Protoperidinium obtusum* | 110239 | | (Karsten) Parke & Dodge, 1976 |
| *Protoperidinium ovatum* | 110241 | | Pouchet, 1883 |
| *Protoperidinium steinii* | 110257 | | (Jörgensen) Balech, 1974 |
| *Protoperidinium pyriforme* | 110249 | | (Paulsen) Balech, 1974 |
| *Pyrophacus horologicum* | 110266 | | Stein, 1883 |
| Unidentified Peridiniales (small) | 109394 | |  |
| Unidentified Peridiniales (large) | 109394 | |  |
| *Torodinium teredo* | 109890 | | (Pouchet) Kofoid & Swezy, 1921 |
| *Torodinium robustum* | 109889 | | Kofoid & Swezy, 1921 |
| *Warnowia* | 109491 | | Lindemann, 1928 |
| Zoospore | N/A | |  |
| *Preperidinium* | 109505 | | Mangin, 1913 |
| TOTAL CILIATES | 11 | |  |
| *Strombidium (small)* | 101195 | | Claparède & Lachmann, 1859 |
| *Strombidium (medium)* | 101195 | | Claparède & Lachmann, 1859 |
| *Strombidium (large)* | 101195 | | Claparède & Lachmann, 1859 |
| *Tontonia ovalis* | 427744 | | (Leegaard, 1915) Laval-Peuto & Rassoulzadegan, 1987 |
| *Strobilidium* | 101185 | | Schewiakoff, 1892 |
| *Mesodinium (small)* | 179320 | | von Stein |
| *Mesodinium rubrum* | 232069 | | (Lohmann, 1908) |
| *Askenasia stellaris* | 292898 | | Leegaard, 1920 |
| *Balanion* | 292899 | | Wulff, 1919 |
| *Didinium* | 341301 | | Stein, 1859 |
| *Strombidinopsis* | 101198 | | Kent, 1881 |
| *Laboea strobila* | 101264 | | Lohmann, 1908 |
| *Leegaardiella* | 101179 | | Lynn & Montagnes, 1988 |
| *Lohmanniella* | 101180 | | Leegaard, 1915 |
| *Peritromus* | 172321 | | Stein, 1863 |
| *Prorodontida* | 425488 | | Corliss, 1974 |
| *Rhabdoaskenasia* | 292925 | | Krainer & Foissner, 1990 |
| *Tiarina* | 247913 | | Berg, 1881 |
| *Tontonia* | 101196 | | Fauré-Fremiet, 1914 |
| *Uronema* | 143943 | | Dujardin, 1841 |
| *Ascampbelliella* | 415082 | |  |
| *Epiplocylis undella* | 341668 | | (Ostenfeld & Schmidt) Jörgensen, 1924 |
| *Eutintinnus* | 183543 | | Kofoid & Campbell, 1939 |
| *Salpingella* | 183566 | | Jörgensen, 1924 |
| *Tintinnopsis* | 163780 | | Stein, 1867 |
| *Favella helgolandica* | 292923 | | (Brandt, 1906) Jörgensen, 1924 |
| *Helicostomella* | 172434 | | Jörgensen, 1924 |
| *Parafavella* | 196836 | | Kofoid & Campbell, 1929 |
| *Proplectella* | 292924 | | Kofoid & Campbell, 1929 |
| *Tintinnid (small)* | 732976 | | Kofoid & Campbell, 1929 |
| *Vorticella* | 163573 | | Linnaeus, 1767 |
| Ciliate | 11 | |  |
| Unidentified tintinnid/ciliate | 11 | |  |
| TOTAL ZOOFLAGELLATES | N/A | |  |
| Bodonidae | 22556 | | Bütschli, 1887 |
| Choanoflagellatea | 580116 | |  |
| Colourless Flagellate | N/A | |  |
| Jacoba | 292929 | | (Patterson, 1990) |
| OTHER |  | |  |
| Amoeba | 605376 | |  |
| Radiozoa | 582421 | |  |
