# Supplementary material for: Ocean Net Heat Flux Influences Seasonal to Interannual Patterns of Plankton Abundance
Source: PLoS One. 2014 Jun 11;9(6):e98709. doi: 10.1371/journal.pone.0098709 (PMC4053316; doi:10.1371/journal.pone.0098709)
Supplement: Table S2 — Station L4 Zooplankton Taxa List. (DOCX) [file pone.0098709.s006.docx]

## Table S2: Station L4 Zooplankton Taxa List

| TAXA | WORMS ID |
| --- | --- |
|  |  |
| Total Zooplankton | N/A |
| *Noctiluca scintillans* | 109921 |
| Foraminifera | 1410 |
| Acantharia | 586732 |
| Radiozoa | 582421 |
| Tintinnida | 425497 |
| Anemone larvae | 1292 |
| Total medusae | 1337 |
| *Solmaris corona* (Narcomedusae) | 117947 |
| Total Trachymedusae | 16350 |
| Trachymedusae | 16350 |
| *Aglantha digitale* | 117849 |
| *Liriope tetraphylla* | 117568 |
| Total Hydromedusae | 1337 |
| Hydromedusae unidentified | 1337 |
| Planula larvae | 1337 |
| Polyp | 1337 |
| Actinula larvae | 1337 |
| *Aurelia aurita* ephyrae | 135306 |
| Scyphozoan ephyrae | 135220 |
| *Amphinema* spp | 117178 |
| *Bougainvillia muscus* | 117328 |
| *Clytia hemisphaerica* | 117368 |
| *Corymorpha nutans* | 117452 |
| *Coryne prolifer* | 231693 |
| *Cosmetira pilosella* | 117747 |
| *Eirene viridula* | 117512 |
| *Eutima gracilis* | 117515 |
| *Hydractinia borealis* | 151761 |
| *Leukartiara octona* | 117791 |
| *Lizzia blondina* | 117345 |
| *Lovenella clausa* | 117736 |
| *Mitrocomella brownei* | 117754 |
| *Obelia* spp. | 117034 |
| *Phialella quadrata* | 117804 |
| *Podocoryne hartlaubi* |  |
| *Rathkea octopunctata* | 117848 |
| *Sarsia prolifera* |  |
| *Sarsia* spp. | 117070 |
| *Slobberina halterata* | 117475 |
| *Turritopsis nutricula* | 117440 |
| *Zanclea costata* | 117998 |
| Total Siphonophore | 1371 |
| *Muggiaea kochi* (polygastric) | 135444 |
| *Muggiaea atlantica* (polygastric) | 135441 |
| *Muggiaea* sp. (eudoxid) | 135366 |
| Siphonophore unidentified | 1371 |
| *Nanomia cara* (nectophore) | 135496 |
| *Agalma elegans* (nectophore) | 135484 |
| Nematoda | 799 |
| Flatworm larvae (Platyhelminth) | 793 |
| *Kuhnia scombr*i eggs (Trematoda) | 119723 |
| Total Ctenophora | 163921 |
| Ctenophore unidentified | 163921 |
| *Pleurobrachia pileus* | 106386 |
| Polychaete larvae unidentified | 883 |
| *Tomopteris helgolandica* | 334946 |
| Total Chaetognath | 2081 |
| Chaetognath eggs | 2081 |
| Chaetognath unidentified | 2081 |
| *Parasagitta elegans* | 105440 |
| *Parasagitta setosa* | 105443 |
| Phoronida actinotroch larvae | 1789 |
| Bryozoa cyphonautes larvae | 146142 |
| Nemertea pilidium larvae | 152391 |
| Gastropod larvae | 101 |
| *Limacina retroversa* | 140227 |
| Bivalvia | 105 |
| *Lamellaria* echinospira larvae | 138101 |
| Gymnosome larvae | 164 |
| *Clione* | 137793 |
| Cephalopoda larvae | 11707 |
| Total Echinodermata | 1806 |
| Echinoderm larvae unidentified | 1806 |
| Ophiopluteus larvae | 123084 |
| Ophiuroid juvenile | 123084 |
| Echinopluteus larvae | 123082 |
| Echinoid Juvenile (Sea urchin larvae) | 123082 |
| Asterioid bipinnaria/brachiolaria | 123080 |
| Asteroid juvenile | 123080 |
| *Luidia* sp. larvae | 123260 |
| Auricularia larvae (Holothuria) | 123083 |
| Doliolaria larvae (Holothuria) | 123083 |
| Tornaria larvae (Hemichordata) | 1818 |
| *Branchiostoma* (Cephalochordata) | 104902 |
| Ascidian tadpole | 1839 |
| Doliolida | 137212 |
| Appendicularia | 146421 |
| Total Fish Eggs | 11676 |
| Sardine eggs (Clupeidae) | 125464 |
| Fish eggs | 11676 |
| Fish larvae | 11676 |
| Cirripede nauplii | 1082 |
| Rhizocephalan nauplii | 1109 |
| Cirripede cyprid | 1082 |
| *Evadne* spp. | 106267 |
| *Podon* spp. | 106269 |
| *Penilia avirostris* | 106272 |
| Isopoda | 1131 |
| Gammariida | 236816 |
| Hyperiida | 1205 |
| Caprellida | 196120 |
| Tanaid | 136164 |
| Cumacea | 1137 |
| Mysida | 149668 |
| Euphausid eggs | 110671 |
| Euphausiid nauplii | 110671 |
| Euphausiid calyptopis | 110671 |
| Euphausiid furcilia | 110671 |
| Euphausiid adult | 110671 |
| Total Decapoda | 1130 |
| Decapod larvae unidentified | 1130 |
| Brachyuran larvae | 106673 |
| Porcellanid larvae | 106838 |
| *Callianassa* spp | 107072 |
| Caridea | 107674 |
| *Ebalia* spp | 106889 |
| *Galathea* spp | 106834 |
| *Jaxea nocturna* | 107737 |
| *Necora* spp | 106927 |
| Paguridae | 106738 |
| *Pontophilus* sp | 107011 |
| *Upogebia* spp | 107079 |
| *Metridia lucens* | 104633 |
| Total *Acartia clausi* | 149755 |
| *Acartia clausi* | 149755 |
| *Acartia clausi* (1-5) | 149755 |
| *Acartia clausi* (female) | 149755 |
| *Acartia clausi* (male) | 149755 |
| *Candacia armata* | 104474 |
| *Centropages chierchiae* | 104494 |
| *Centropages hamatus* | 104496 |
| Total *Centropages typicus* | 104499 |
| *Centropages typicus* | 104499 |
| *Centropages* spp (1-5) | 104159 |
| *Centropages typicus* (female) | 104499 |
| *Centropages typicus* (male) | 104499 |
| *Isias clavipes* | 104501 |
| *Anomalocera patersoni* | 104722 |
| *Parapontella brevicornis* | 104686 |
| *Eurytemora affinis* | 104872 |
| *Labidocera wollastoni* | 104736 |
| Total *Temora longicornis* | 104878 |
| *Temora longicornis* | 104878 |
| *Temora longicornis* (1-5) | 104878 |
| *Temora longicornis* (female) | 104878 |
| *Temora longicornis* (male) | 104878 |
| *Temora stylifera* | 104879 |
| *Calanoides carinatus* | 104462 |
| Total *Calanus helgolandicus* | 104466 |
| *Calanus helgolandicus* | 104466 |
| *Calanus* copepodites (1-5) | 104152 |
| *Calanus helgolandicus* (female) | 104466 |
| *Calanus helgolandicus* (male) | 104466 |
| *Calanus* eggs | 104152 |
| *Calanus finmarchicus* female | 104464 |
| *Calocalanus* spp. | 104193 |
| Total *Clausocalanus* spp. (Calculated) | 104161 |
| *Total Ctenocalanus vanus (Calculated)* | 104510 |
| *Total Paracalanus parvus (Calculated)* | 104685 |
| *Total Pseudocalanus elongatus (Calculated)* | 104515 |
| *Para/Pseudo/Cteno/Clausocalanus* Unidentified (1-5) | 1100 |
| *Clausocalanus* spp. (female) | 104161 |
| *Clausocalanus* spp. (male) | 104161 |
| *Ctenocalanus vanus* (female) | 104510 |
| *Ctenocalanus vanus* (male) | 104510 |
| *Paracalanus parvus* (female) | 104685 |
| *Paracalanus parvus* (male) | 104685 |
| *Pseudocalanus elongatus* (female) | 104515 |
| *Pseudocalanus elongatus* (male) | 104515 |
| *Subeucalanus* spp. | 104173 |
| *Microcalanus* spp. | 104164 |
| *Diaixis hibernica* | 104521 |
| *Paraeuchaeta hebes* | 104563 |
| *Scolecithricella* spp. | 104229 |
| *Oithona* spp. | 106485 |
| *Oncaea* spp. | 128690 |
| *Ditrichocorycaeus anglicus* | 128805 |
| *Microsetella* sp | 115341 |
| *Euterpina acutifrons* | 116162 |
| *Goniopsyllus clausi* | 346509 |
| Monstrilloida | 1106 |
| Harpacticoid unidentified | 1102 |
| Siphonostomatoida | 1104 |
| Copepod nauplii | 1080 |
| Acarid mites | 150325 |
